# Primary diffuse large B-cell lymphoma of the right kidney: a case report

**DOI:** 10.11604/pamj.2022.42.269.34470

**Published:** 2022-08-11

**Authors:** Hamid Nasrollahi, Ali Eslahi, Faisal Ahmed, Bita Geramizadeh, Mansour Ansari

**Affiliations:** 1 Radiation Oncology Department, School of Medicine, Shiraz University of Medical Sciences, Shiraz, Iran,; 2 Department of Urology, School of Medicine, Shiraz University of Medical Sciences, Shiraz, Iran,; 3 Urology Research Center, Al-Thora General Hospital, Department of Urology, School of Medicine, Ibb University of Medical Sciences, Ibb, Yemen,; 4 Department of Pathology, School of Medicine, Shiraz University of Medical Sciences, Shiraz, Iran

**Keywords:** Diffuse large B-cell lymphoma, primary renal lymphoma, case report

## Abstract

The existence of primary renal lymphoma (PRL) in the kidney has long been debated due to its extranodal location and lack of lymphatic channels. Primary renal lymphoma is extremely rare, accounting for less than 1%, and is frequently misdiagnosed as renal cell carcinoma (RCC). We present a 50-year-old man presenting with right flank pain in the last week. The computed tomography scan showed a large isodense right renal mass with a small para-aortic lymph node suspected of RCC. The patient underwent right radical nephrectomy and lymphadenectomy with an uneventful postoperative outcome. The histopathology and immunohistochemistry showed diffuse large B-cell lymphoma. Then, the patient received five-cycle chemotherapy and regional radiotherapy. Within five years of follow-up, no symptoms of recurrence. In conclusion, even though PRL is a rare tumor type. An effort should be made to make a preoperative diagnosis because PRL can be treated with systemic chemotherapy instead of other renal tumors requiring nephrectomy.

## Introduction

Systemic non-Hodgkin's lymphoma (NHL) involving the kidneys is common (30-60%); however, primary isolated lymphoma of the kidney is sporadic, with about <1% of all renal tumors and diffuse large B cell lymphoma (DLBCL) representing the most dominant pathological histotype [1,2]. Primary renal lymphoma (PRL) is frequently misdiagnosed as renal cell carcinoma (RCC) [3]. Patients with PRL may exhibit nonspecific symptoms and signs, such as flank pain, weight loss, pyrexia, hematuria, and palpable mass [3]. Only a few cases of PRL have been reported [4]. Hence, we present a case of PRL in a 50-year-old man who presented with right flank pain and discussed its clinical manifestations, treatment, and outcome.

## Patient and observation

**Patient information:** we present a 50-year-old man who came to the clinic with right flank pain radiating to the abdomen in the last week. The patient has no history of hematuria, fever, weight loss, night sweats, persistent fatigue, loss of appetite, cough, chest pain, or stomach pain. No familial history of infectious, malignancy, or genetic disease.

**Clinical findings:** in the physical exam, the patient has a large non-mobile right flank mass with mild tenderness in this zone.

**Figure 1:**
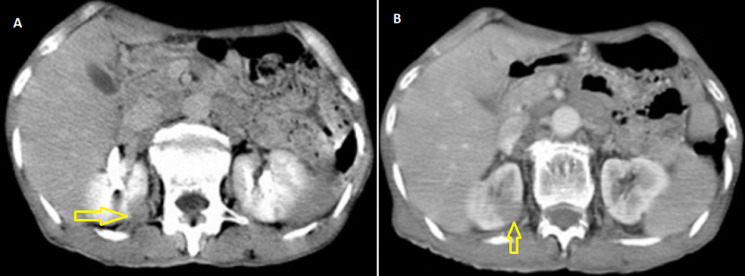
A) abdominal CT scan showing a right renal mass (arrow); B) low significant enhancement (arrow)

**Diagnostic assessment:** laboratory results were as follows: white blood cell: 7 ×103/ml, hemoglobin: 14.4 g/dl, blood urea nitrogen: 14 mg/dl, and creatinine: 1.1 mg/dl. Urine analysis showed microscopic hematuria (15-20 RBCs/HPF). Other blood tests were within normal range. The abdominal ultrasonography showed a mildly enlarged right kidney with heterogeneous hypoechoic mass measuring 70 x 63 mm in mid and upper poles suggestive of malignant lesion. The abdominal computed tomography (CT) scan showed a large isodense right renal mass entirely occupying the renal parenchyma with extension to the renal pelvis and upper ureter without significant enhancement measuring 90 x 80 mm and without involvement of renal vessels. There are a few small lymph nodes in the right para-aortic area less than 10 mm (Figure 1). The tumor stage was T3b N1 (involvement of local lymph nodes) [5].

**Therapeutic interventions:** with suspicion of RCC, after general anesthesia and with a right subcostal incision, the retroperitoneal space was opened. A large renal mass and some enlarged regional lymph nodes were identified. After mobilization and release of the right kidney, right radical nephrectomy and regional lymph node dissection were performed.

**Figure 2:**
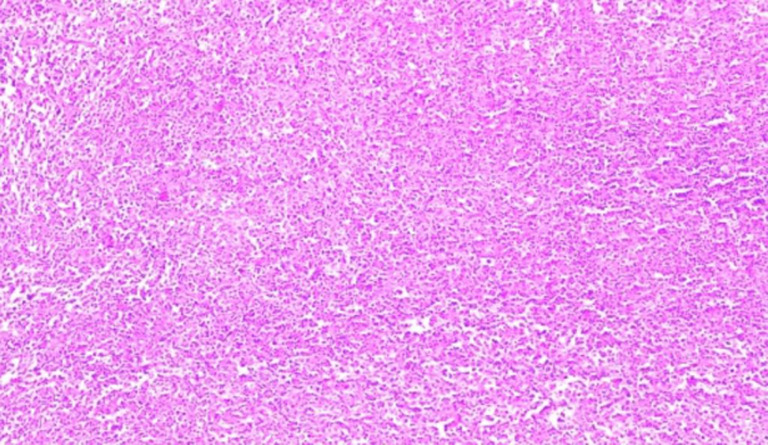
histopathology of right renal mass showing diffuse infiltration of atypical lymphocyte (H&E x250)

**Figure 3:**
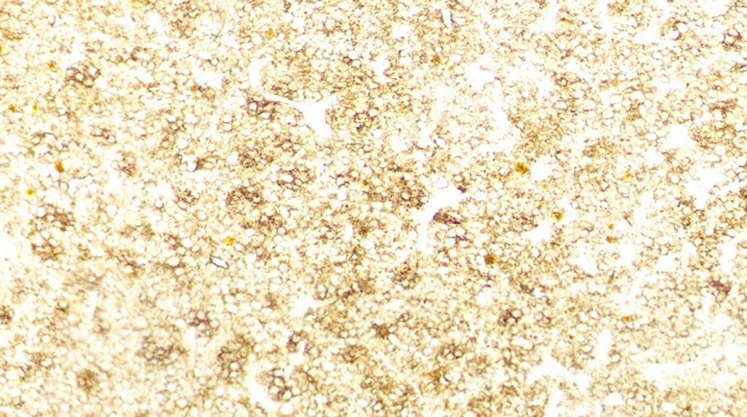
immunohistochemistry staining of the resected mass showing diffuse and strongly positive for CD20 (H&E x250)

**Follow-up and outcome:** the postoperative follow-up period was uneventful, and the patient was discharged on the fourth postoperative day. The histopathology report showed lymphoma cells that were small and in a diffuse pattern (Figure 2). The immunohistochemistry (IHC) study was positive for CD20, CD45, Ki-67 (70%) and negative for CD10, CD5, and CK1, suggestive of the diagnosis of DLBCL (Figure 3). The patient was referred to an oncologist for further evaluation. Bone marrow biopsy and chest CT did not show any evidence of systemic lymphoma invasion. Patient was not able to pay for PET-CT scan. The patient received the R-CHOP regimen (rituximab, cyclophosphamide, doxorubicin, vincristine, and prednisolone) for six standard courses with regional radiotherapy (dose of 30 Gy in ten fractions). According to standard guidelines, routine follow up was performed. Physical examination and history taking at each visit was done. Chest X-rays and lab tests were checked annually. Within five years of follow-up, there were no recurrences or metastases.

**Patient perspective:** the patient was happy with the successful outcome of the surgery.

**Informed consent:** a written informed consent was obtained from the patient for participation in our study.

## Discussion

Primary renal lymphoma (PRL) is a rare malignant tumor that accounts for less than 1% of all renal masses [6]. It is more common in men than women within an age range of 45-65 years, and bilateral renal involvement has been seen in up to 20% of patients [7]. Similarly, our patient is a 50-year-old man. Because the kidney is an extranodal organ with no lymphatic channels, the etiology of PRL is unknown. Although it is unknown how lymphoma develops in the kidneys, several theories explain this occurrence. Some experts suggest that PRL begins in the lymphatics of the renal capsule and spreads to the renal parenchyma [8]. Others suggest the possible hematogenous spread of the disease [4]. The kidney's mucosa-associated lymphoid tissue (MALT) is also a source site for PRL [8]. Due to its rarity and unknown etiology, PRL is usually ignored in the differential diagnosis of renal masses, favoring more prevalent malignancies such as RCC and benign renal cysts [6-8]. Similarly, our case was first operated with a high suspicion of RCC. DLBCL is the most common type of PRL, followed by lymphomas of the extranodal marginal zone [7]. Other B-cell lymphomas, such as small lymphocytic lymphoma, chronic lymphocytic leukemia, and Burkitt lymphoma, represented less than 5% of PRL cases [7].

The symptoms of PRL are similar to other renal masses and include hematuria, flank pain, and a palpable mass on the flank or abdomen [9]. Additionally, patients may present with B symptoms (fever, night sweats, and weight loss) typically seen in lymphomas [10]. Our patient was suffering from abdominal pain. RCC has a more heterogeneous appearance on radiologic imaging than PRL. However, the CT scan findings are not specific, making the differentiation much more difficult [6]. Atypical findings on CT scan, such as bilateral renal mass, homogeneous renal enlargement without palpable mass, multiple renal nodules, or hypovascularity, may be considered an indication for fine-needle renal biopsy before surgery [6,11]. Positron emission tomography (PET) scan can help with diagnosis, clinical staging, prognosis, and treatment. It can detect metabolic activity and determine its involvement [12]. PET/CT was not performed in our case as we highly suspect RCC.

Radical nephrectomy is the standard treatment for suspected renal masses. However, PRL is usually managed by neoadjuvant chemotherapy prior to surgery [6]. As a result, exact diagnosis and confirmation are critical to distinguish RCC from PRL. Early diagnosis and treatment with (R-CHOP) can improve renal function within 2 to 4 weeks [6,9]. The 1-year mortality rates of PRL can reach 75% despite oncological treatment, with a five-year survival rate of only 40-50% [8,13]. To minimize chemotherapy exposure and toxic effect in patients with early-stage, low-risk DLBCL, three cycles R-CHOP followed by field radiation therapy is a well-established and popular treatment modality [14]. Our patient received five cycles of chemotherapy (R-CHOP) and regional radiotherapy with a good survival.

Pfreundschuh *et al*. on the other hand, reported that six cycles of R-CHOP every 21 days are adequate without radiation therapy in younger patients with DLBCL with favorable features (early-stage DLBCL and no tumor bulk). The progression-free survival rate was 89.6 % at six years, and the overall survival rate was 94.9% [15]. Chen *et al*. described a 70-year-old woman diagnosed with PRL after undergoing radical nephrectomy. After 6-8 cycles of (R-CHOP), there was no evidence of local recurrence after two months of follow-up. Additionally, the authors examined 49 cases of PRL diagnosed since 1989. They mentioned that bilateral PRL was associated with a shorter survival rate than unilateral PRL (21 vs 68 months), and utilizing chemotherapy alone was associated with a shorter survival rate than those who received combination chemotherapy and surgery (15.8 vs 49.4 months) [16].

Taneja *et al*. studied all patients between 1973 and 2015 and found that the median overall survival was 112 months, but the median cause-specific survival was not achieved. They stated that being over 60 was the most potent independent risk factor for poor overall survival and cause-specific survival, whereas non-DLBCL histology was associated with better overall survival and cause-specific survival [7]. Our patient had a good outcome, and within five years of follow-up, no recurrence was detected. In DLBCL, the histological picture is either a dense interstitial infiltrate of lymphoma cells with an expanded interstitium or glomerular infiltration that can be confused with pseudo-proliferative glomerulonephritis [17]. The IHC study is the gold standard for diagnosis and treatment. IHC for DLBCL is generally positive for B-cell markers such as CD20, CD21, CD97, and CD45, which were similar in our case [9].

Finally, we emphasize that surgeons should pay more attention to preoperative diagnosis and avoid unnecessary radical nephrectomy, as patients with PRL can have a good outcome after standardized and systematic chemotherapy.

## Conclusion

Even though PRL is a rare tumor type, it should be considered when making a differential diagnosis of any renal mass. A high index of suspicion for PRL should be maintained if there is lymphadenopathy or atypical findings on radiology images. An effort should be made to make a preoperative diagnosis because PRL can be treated with systemic chemotherapy instead of other renal tumors requiring nephrectomy.
